# Nb^5+^-Doped SrCoO_3−*δ*_ Perovskites as Potential Cathodes for Solid-Oxide Fuel Cells

**DOI:** 10.3390/ma9070579

**Published:** 2016-07-15

**Authors:** Vanessa Cascos, José Antonio Alonso, María Teresa Fernández-Díaz

**Affiliations:** 1 Instituto de Ciencia de Materiales de Madrid, CSIC, Cantoblanco, 28049 Madrid, Spain; jaalonso@icmm.csic.es; 2 Institut Laue Langevin, BP 156X, 38042 Grenoble, France; ferndiaz@ill.fr

**Keywords:** SrCoO_3−*δ*_, SOFC, cathode, hydrogen, SrCo_1−*x*_Nb*_x_*O_3−_*_δ_*, solid oxide fuel cell, neutron diffraction

## Abstract

SrCoO_3−*δ*_ outperforms as cathode material in solid-oxide fuel cells (SOFC) when the three-dimensional (3C-type) perovskite structure is stabilized by the inclusion of highly-charged transition-metal ions at the octahedral positions. In a previous work we studied the Nb incorporation at the Co positions in the SrCo_1−*x*_Nb*_x_*O_3−*δ*_ system, in which the stabilization of a tetragonal *P4*/*mmm* perovskite superstructure was described for the *x* = 0.05 composition. In the present study we extend this investigation to the *x* = 0.10–0.15 range, also observing the formation of the tetragonal *P4*/*mmm* structure instead of the unwanted hexagonal phase corresponding to the 2H polytype. We also investigated the effect of Nb^5+^ doping on the thermal, electrical, and electrochemical properties of SrCo_1−*x*_Nb*_x_*O_3−*δ*_ (*x* = 0.1 and 0.15) perovskite oxides performing as cathodes in SOFC. In comparison with the undoped hexagonal SrCoO_3−*δ*_ phase, the resulting compounds present high thermal stability and an increase of the electrical conductivity. The single-cell tests for these compositions (*x* = 0.10 and 0.15) with La_0.8_Sr_0.2_Ga_0.83_Mg_0.17_O_3−*δ*_ (LSGM) as electrolyte and SrMo_0.8_Fe_0.2_CoO_3−*δ*_ as anode gave maximum power densities of 693 and 550 mW∙cm^−2^ at 850 °C respectively, using pure H_2_ as fuel and air as oxidant.

## 1. Introduction

Among the different types of fuel cells, SOFC (solid oxide fuel cells) are well-known to exhibit the highest energy conversion rates, associated with the elevated operation temperatures of these devices (typically 1000 °C), where all the components are metal oxides [1]. The development of oxides with mixed ionic-electronic conductivity (MIEC) has been crucial for the decrease of the operation temperature, without a significant drop of the performance of the cells. In fact, the cathode is responsible for a considerable reduction in the cell potential in a SOFC at intermediate temperatures (IT, 650–850 °C). The forefront researches on SOFC cathodes with MIEC properties include perovskite structures that combine a high tolerance to oxygen vacancies, strong metal-oxygen-metal covalent bonds to get the bandwidth required for electronic conductivity and a good activity for oxygen reduction.

SrCoO_3−*δ*_–based perovskites have been extensively used as MIEC cathodes in IT-SOFC [2,3,4]. Ba_0.5_Sr_0.5_Co_0.8_Fe_0.2_O_3−*δ*_ (BSCF) [5] and SrCo_0.8_Fe_0.2_O_3−*δ*_ (SCF) perovskites have been especially investigated because they exhibit high ionic conductivity values and a good catalytic activity above 600 °C [6,7]. However, these materials exhibit a moderate chemical stability, which may limit the performance after long operation times [8,9].

In fact, SrCoO_3−*δ*_ itself is well-known to exhibit one of the highest electrical conductivities and oxygen permeation fluxes [10,11,12], when stabilized in its cubic form. Unhappily, this cubic polymorph is not stable below 900 °C where a 3C-cubic to 2H-hexagonal phase transition takes place when the material undergoes a slow cooling process. Therefore, the stabilization of a 3C-type perovskite framework (consisting of an arrangement of CoO_6_ octahedra sharing corners across the three crystallographic directions) in the SrCoO_3−*δ*_ system has been a widely used strategy to obtain an adequate mixed ionic-electronic conductor to be used as a cathode in IT-SOFC. It has been found that partial atomic replacements with aliovalent elements either at the Sr position or the Co positions led to the stabilization of the wanted 3C arrangement, in perovskite superstructures that performs well as cathodes with very low polarization resistance at intermediate temperatures [13,14,15,16,17].

A successful way to stabilize the 3C polymorph of SrCoO_3−*δ*_ in a tetragonal perovskite superstructure at room temperature is the introduction of a low amount of a highly charged cation such as Sb, Mo or Sn [15,16,17] at the Co positions in order to avoid the formation of the unwanted 2H-hexagonal structure. The inclusion of a highly charged cation at the octahedral positions induces electrostatic repulsion effects that destabilize the octahedral face-sharing configuration present in the 2H hexagonal phase.

Following this strategy, in a previous work we studied the Nb incorporation at the Co positions in the SrCo_1−_*_x_*Nb*_x_*O_3−_*_δ_* system, in which the stabilization of a tetragonal *P4*/*mmm* perovskite superstructure was described for the *x* = 0.05 composition [18]. In the present work we extended the Nb contents to 10% and 15%, also obtaining a tetragonal perovskite at room temperature. We investigated the effect of Nb-doping on the crystal structure and the thermal, electrical, and electrochemical properties, finally showing that these oxides can be successfully used as cathodes in SOFC using H_2_ as fuel. The material characterization includes a neutron power diffraction (NPD) study for *x* = 0.10 from which interesting structural features are unraveled, in complement with dilatometry analysis, thermal measurements, electronic conductivity measurements, chemical stability with the electrolyte, and single-cell tests in real SOFC devices.

## 2. Experimental

SrCo_0.90_Nb_0.10_O_3−*δ*_ and SrCo_0.85_Nb_0.15_O_3−*δ*_ perovskites were prepared as polycrystalline powders by a citrate method. Stoichiometric amounts of Sr(NO_3_)_2_ and Co(NO_3_)_2_∙6H_2_O were dissolved in citric acid with a few drops of HNO_3_, and a stoichiometric amount of Nb_2_O_5_ powder was later added and held in suspension with continuous stirring; the suspension was slowly evaporated and the resins were decomposed at 600 °C for 12 h. Once this step was completed, the obtained precursors were heated in air at 900 °C for 12 h, followed by a treatment at 1000 °C for 24 h. Additional heating at 1100 °C for 12 h was carried out in order to eliminate or minimize secondary phases as the hexagonal phase.

The initial characterization of the products was carried out by laboratory X-ray diffraction (XRD) using a Bruker D8 diffractometer (40 kV, 30 mA) (Madrid, Spain), controlled by a DIFFRACT^PLUS^ software (Bruker Española S.A., Madrid, Spain), in Bragg-Brentano reflection geometry with CuKα radiation (λ = 1.5418 Å) and a PSD (Position Sensitive Detector). The data were obtained between 10° and 64° in steps of 0.05°. For the structural refinement of SrCo_0.9_Nb_0.1_O_3−*δ*_, a NPD pattern was collected at the D2B diffractometer of the Institut Laue-Langevin, Grenoble, with a wavelength λ = 1.594 Å within the 2θ range from 10° to 154° at 25 °C. About 2 g of sample was contained in a vanadium can; a time of 2 h was required to collect a full diffraction pattern. The NPD data were analyzed by the Rietveld method [19] with the FULLPROF program [20]. A pseudo-Voigt function was chosen to generate the line shape of the diffraction peaks. In the final run, the following parameters were refined: scale factor, background points, zero shift, half-width, pseudo-Voigt corrected for asymmetry parameters, unit-cell parameters, positional coordinates, isotropic displacement factors for the metals, and anisotropic for the oxygen atoms. The coherent scattering lengths of Sr, Co, Nb, and O were 7.02, 2.49, 7.054, and 5.803 fm, respectively.

Thermal analysis was performed in a Mettler TA3000 system equipped with a TC10 processor unit (Mettler Toledo, Madrid, Spain). Thermogravimetric (TG) curves were obtained in a TG50 unit, working either in air flow at a heating rate of 10 °C·min^−1^ using about 50 mg of sample in each experiment.

Measurements of the thermal expansion coefficient required the use of sintered cylindrical samples (5 mm diameter × 2 mm thickness) whereas the electrical conductivity was carried out in sintered bar-shaped samples (3 × 3 × 10 mm^3^). Densification was performed by uniaxial pressing of the pellets that were subsequently calcined at 1100 °C for 12 h; the obtained density was around 80%–85% of the theoretical one. Thermal expansion of the sintered samples was performed in a dilatometer Linseis L75HX1000 (Linseis Thermal Analysis, Madrid, Spain), between 100 and 850 °C in air atmosphere. The conductivity was measured between 25 and 850 °C in air by the four-point method under *dc* currents between 0.1 and 0.5 A. The currents were applied and collected with a Potentiostat-Galvanostat AUTOLAB PGSTAT 302 (Metrohm Autolab B.V., Madrid, Spain) from ECO CHEMIE.

Single-cell tests were carried out using LSGM pellets as electrolyte, SrCo_1−*x*_Nb*_x_*O_3−*δ*_ (SCNO) (*x* = 0.1 and 0.15) as cathode material, and SrMo_0.8_Fe_0.2_O_3−*δ*_ (SMFO) as anode material, recently developed in our group [21]. La_0.4_Ce_0.6_O_2−*δ*_ (LDC) was used as a buffer layer between the anode and the electrolyte in order to prevent the interdiffusion of ionic species. Inks of LDC, SMFO, and SCNO were prepared with a binder (V-006 from Heraeus, Madrid, Spain). LDC ink was screen-printed onto one side of the LSGM disk followed by a thermal treatment at 1300 °C in air for 1 h. SMFO was subsequently screen printed onto the LDC layer and fired at 1100 °C in air for 1 h. SCNO was finally screen printed onto the other side of the disk and fired at 1100 °C in air for 1 h. The working electrode area of the cell was 0.25 cm^2^ (0.5 × 0.5 cm^2^). Pt gauze with a small amount of Pt paste was used as current collector at both the anodic and the cathodic sides for ensuring electrical contact. The cells were tested in a vertical tubular furnace at 800 °C and 850 °C; the anode side was fed with pure dry H_2_, whereas the cathode worked in an air flow. The fuel-cell tests were performed with an AUTOLAB 302N Potentiostat/Galvanostat (Metrohm Autolab B.V., Madrid, Spain) by changing the voltage of the cell from the OCV (“Open current voltage”) to 0.1 V, in steps of 0.010 V, holding 10 seconds at each step. Current density was calculated by the recorded current flux through the effective area of the cell (0.25 cm^2^).

## 3. Results and Discussion

### 3.1. Crystallographic Characterization

**Figure 1:**
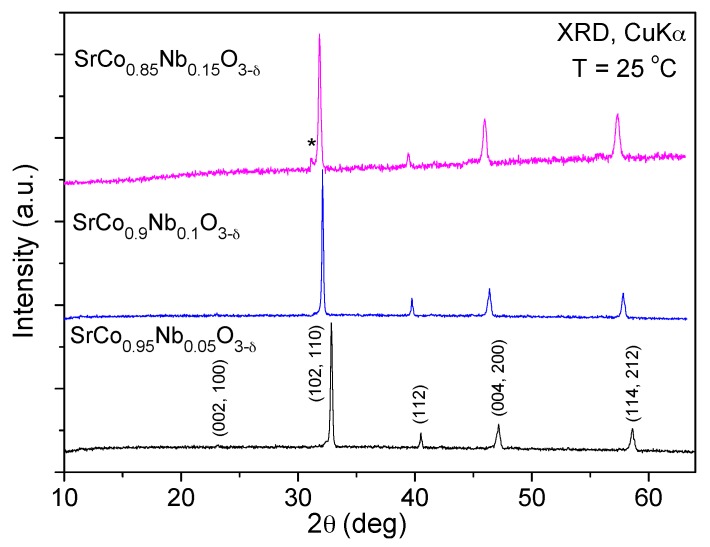
X-ray diffraction (XRD) patterns (Cu K_α_) for SrCo_1−*x*_Nb*_x_*O_3−*δ*_ (*x* = 0.05, 0.10, and 0.15) indexed in the tetragonal *P4*/*mmm* space group. *x* = 0.05 data are taken from ref. [18], reprinted and adapted from [18], with permission from © 2014 Elsevier.

SrCo_1−*x*_Nb*_x_*O_3−*δ*_ oxides were obtained as well crystallized perovskite phases; Figure 1 shows the XRD patterns at room temperature for SrCo_1−*x*_Nb*_x_*O_3−*δ*_ (*x* = 0.05, 0.10, and 0.15), which are indexed on the basis of a tetragonal perovskite unit cell with a = b~*a*_o_, c~2*a*_o_. The XRD pattern for *x* = 0.05 has been taken from ref. [18]. The material with *x* = 0.15 was obtained with a small impurity of the hexagonal phase as shown in Figure 1, with an asterisk for the most intense reflection.

Table 1 contains the unit-cell parameters and discrepancy factors after the Rietveld refinements of the structures studied by X-ray diffraction at room temperature, compared with those corresponding to the *x* = 0.05 oxide from ref. [18]. The unit-cell parameters of the SrCo_1−*x*_Nb*_x_*O_3−*δ*_ (*x* = 0.05, 0.10 and 0.15) series increase with the amount of Nb, as shown in Table 1; this fact may be ascribed, in principle, to the larger ionic size for Nb^5+^ ions (0.64 Å) with respect to both Co^3+^ (0.61 Å) and Co^4+^ (0.53 Å) [22].

**Figure 2:**
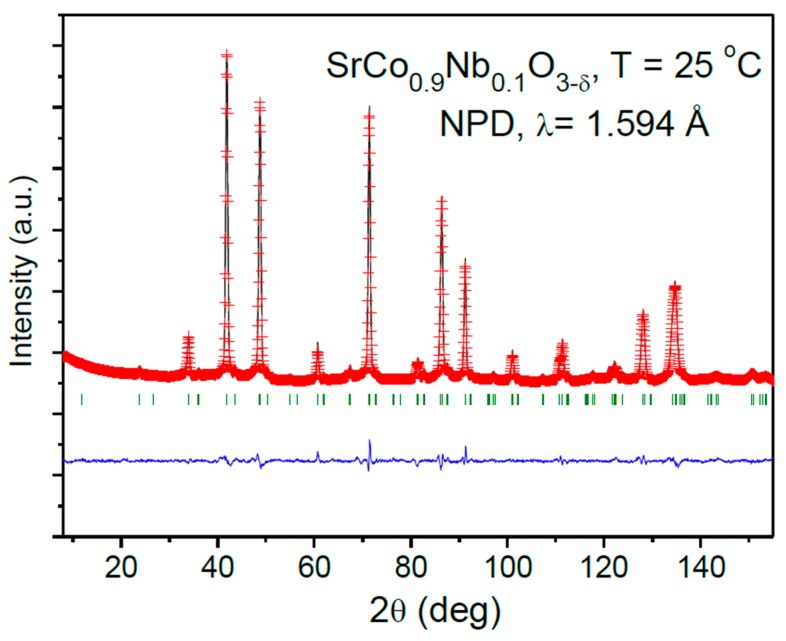
Observed (red crosses), calculated (black line) and difference (bottom line) neutron power diffraction (NPD) Rietveld profiles for SrCo_0.9_Nb_0.1_O_3−*δ*_. The series of Bragg markers corresponds to the crystallographic structure.

The crystal structure of SrCo_0.9_Nb_0.1_O_3−*δ*_ perovskite has been refined from NPD data considering the same superstructure described in ref. [18], defined in a tetragonal unit cell with a doubled axis along the *c* direction, in the *P4*/*mmm* (n° 123) space group with Z = 2. In this perovskite, Sr atoms are located at 2*h* (1/2, 1/2, *z*) sites, two non-equivalent Co atoms are placed at 1*a* (0, 0, 0) (Co1) and 1*b* (0, 0, 1/2) (Co2), Nb atoms are distributed at 1*b* (0, 0, 1/2) together with Co2 (called (Co,Nb)2), and three oxygen atoms (O1, O2, O3) are located at 2*f* (1/2, 0, 0), 2*g* (0, 0, *z*) and 2*e* (1/2, 0, 1/2) Wyckoff positions, respectively. The occupancy factors for oxygen atoms were also refined, as well as the anisotropic displacement factors for all the oxygen positions. It was found that most of the vacancies are concentrated at O3 site (f_occ_ = 0.829(1)) showing a high equivalent isotropic factor of 3.07 Å^2^. It was also found that Nb^5+^ ions are all placed at 1*b* positions and the mixed Co/Nb occupancy factor is very close to that expected from the nominal stoichiometry by contrast with SrCo_0.95_Nb_0.05_O_3−*δ*_ in which a little amount of Nb was also found at 1*a* position. After the complete NPD refinement we obtained the crystallographic formula Sr(Co_0.911(1)_Nb_0.089(1)_)O_2.829(1)_. Figure 2 illustrates the goodness of the fit after the Rietveld refinement from NPD data for SrCo_0.9_Nb_0.1_O_3−*δ*_; Table 2 lists the final structural parameters, selected atomic distances and discrepancy factors for SrCo_1−*x*_Nb*_x_*O_3−*δ*_ (*x* = 0.15 and 0.1). The minor difference between the unit-cell parameters obtained from XRD and NPD come from a slight inaccuracy of the neutron wavelength.

**Figure 3:**
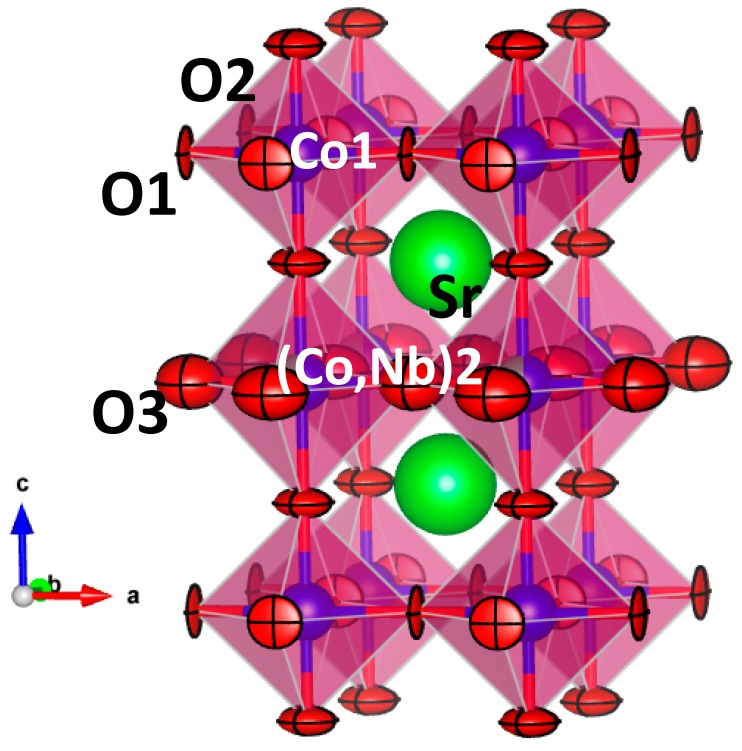
Tetragonal perovskite superstructure observed for SrCo_0.9_Nb_0.1_O_3−*δ*_ from NPD data at RT.

The crystal structure of SrCo_0.9_Nb_0.1_O_3−*δ*_ at room temperature is shown in Figure 3. It is a tetragonal perovskite superstructure consisting of two cubic aristotypes stacked along the *c* direction. As mentioned, Nb atoms are randomly distributed at Co2 positions, whereas Co1 sites do not apparently contain Nb. This superstructure probably arises from the long-range ordering of oxygen vacancies, since they are all concentrated at the O3 position. Both O1 and O2 oxygen sites are stoichiometric within the standard deviations. The oxygen vacancies belong, therefore, to the coordination polyhedron of (Co,Nb)2. It is noteworthy that (Co,Nb)2O_6_ octahedra are significantly elongated, with four equatorial (Co,Nb)2-O3 bond lengths of 1.92964(3) Å and two particularly long axial (Co,Nb)2-O2 distances of 2.032(4) Å. Correspondingly, the Co1 octahedra are flattened along the *c* axial direction, displaying extremely short Co1-O2 distances of 1.840(4) Å. The four equivalent equatorial distances are also 1.92964(3) Å. This long-range arrangement could be the second ingredient of the observed superstructure along the *c* axis. This phenomenology is reminiscent of that described for SrCo_1−*x*_M*_x_*O_3−*δ*_ (M = Ti, V) [23] where these differences in the octahedral size suggested that Co1 cations may present a higher oxidation state than Co2. The average oxidation state for Co is 3.53+ assuming a pentavalent oxidation state for Nb and divalent for Sr. This involves an oxidation state of Co^4+^ in Co1 sites and Co^3+^ in (Co,Nb)2 positions, assuming a full charge disproportionation. In fact, the presence of Co^3+^ with an intermediate-spin in Co2 sites could cause the Jahn-Teller distortion of the Co-O bond lengths, and O^2−^ ions would approach Co^4+^ cations, moving from Co2 to Co1.

The refinement of the anisotropic displacement factors for all the oxygen atoms from NPD data gives clues on the mechanism of oxygen diffusion in this material. Figure 3 shows the displacement ellipsoids for the oxygen atoms, drawn for 95% probability. For O1, with occupancy factors close to unity, the thermal ellipsoids are extremely flattened, and can be described as disks perpendicular to the Co1-O1-Co1 bonding direction. This shape and configuration suggest strongly covalent Co-O-Co chemical bonds, compatible with the high oxidation states previously determined for Co1; the strength of these bonds and the full occupancy of these positions make it improbable that O1 atoms take part from the oxygen diffusion process of this MIEC material. Axial O2 oxygens also show anisotropic ellipsoids, flattened perpendicular to the Co1-O2-(Co,Nb)2 directions; this effect is less patent than for O1, but the shape and orientation of O2 ellipsoids, also fully occupied, suggest as well a meagre contribution to the oxygen conduction process. For O3 (where most of the oxygen deficiency is concentrated) the anisotropy of the thermal ellipsoids is smaller, adopting an almost spherical shape. These are highly deficient sites, with large and almost isotropic displacements that suggest a smearing of the nuclear density as the oxygen atoms diffuse across the three directions of the crystal. Therefore, these atoms are particularly involved in oxygen conductivity.

### 3.2. Thermal Analysis (TGA)

**Figure 4:**
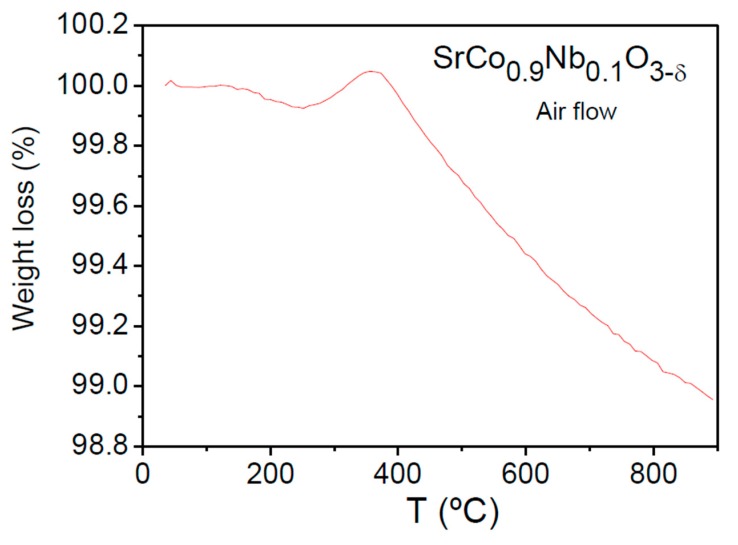
Thermogravimetric analysis (TGA) curve of SrCo_0.9_Nb_0.1_O_3−*δ*_ in air flow.

The oxygen content of the samples was determined by thermogravimetric analysis carried out in air from 25 to 900 °C. Figure 4 shows the TGA curves for the SrCo_0.9_Nb_0.1_O_3−*δ*_ perovskite. It shows a minor weight loss up to 300 °C. A conspicuous weight gain is observed between 300 and 400 °C. This was also previously observed in Sb-doped oxides [17] and it could be related to partial Co oxidation with a slight incorporation of oxygen atoms into the unit cell. From 400 °C, there is a regular and important weight loss up to 900 °C of 1.05% corresponding to 0.13 oxygen atoms per formula. This indicates that, at the working conditions of a SOFC, the cathode material is even more oxygen deficient, which undoubtedly improves the O^2−^ motion of this MIEC oxide.

After the TGA analysis, the XRD pattern of the resulting solid is identical to the starting pattern. This confirms no phase separation or decomposition after heating in air, and complete reversibility of the absorption-desorption oxygen process.

### 3.3. Thermal Expansion Measurements

**Figure 5:**
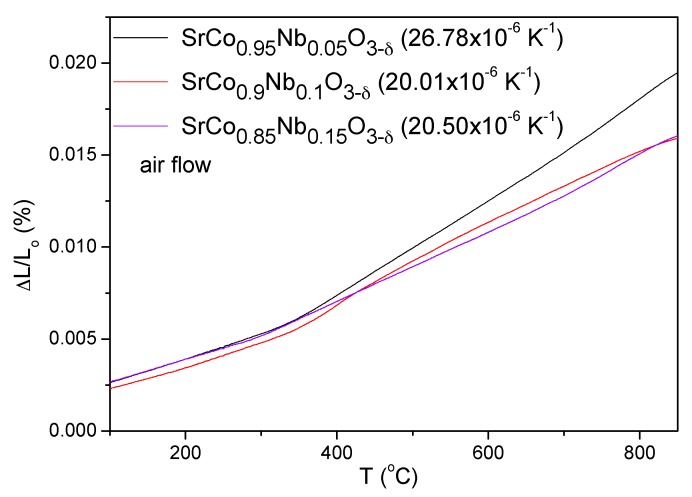
Thermal expansion determined by dilatometry of the SrCo_1−*x*_Nb*_x_*O_3−*δ*_ (*x* = 0.05, 0.1, and 0.15) perovskites (data for *x* = 0.05 are taken from ref. [18], reprinted and adapted from [18], with permission from © 2014 Elsevier).

The thermal expansion coefficients (TECs) of SrCo_1−*x*_Nb*_x_*O_3−*δ*_ (*x* = 0.05, 0.10, and 0.15) were measured in cylindrical dense ceramics (5 mm diameter × 2 mm thickness) sintered at 1100 °C for 12 h. The data for *x* = 0.05 [18] are included for the sake of comparison. A dilatometric analysis was performed between 100 and 850 °C for several cycles; the data were only recorded during the heating runs. Figure 5 shows no abrupt anomalies in the thermal expansion of SrCo_1−*x*_Nb*_x_*O_3−*δ*_ in all the temperature range under measurement; however, all the compounds show a change in slope between 350 and 400 °C which may be related to the weight gain observed in Figure 4 at the same temperature for *x* = 0.1 oxide.

The TEC values for these compounds are included in Figure 5. There is a considerable decrease of the TEC from *x* = 0.05 to *x* = 0.10, which then remains unchanged for *x* = 0.15. Even so, these values are higher than those normally presented by SOFC components but are typical of Co-containing perovskites. These values could be minimized in composites with the electrolyte.

### 3.4. Electrical Conductivity Measurements

**Figure 6:**
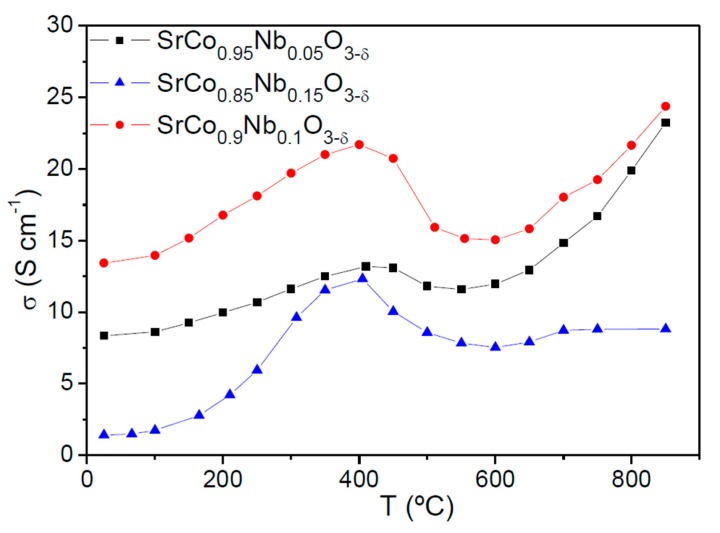
Dc-conductivity as a function of temperature for SrCo_1−*x*_Nb*_x_*O_3−*δ*_ (*x* = 0.05, 0.10, 0.15). Data for *x* = 0.05 are taken from ref. [18], reprinted and adapted from [18], with permission from © 2014 Elsevier.

Figure 6 shows the thermal variation of the electrical conductivity of SrCo_1−*x*_Nb*_x_*O_3−*δ*_ (*x* = 0.05, 0.10, and 0.15) measured in sintered bars (10 mm large × 3 mm width × 3 mm high) in air atmosphere by the *dc* four-probe method; the bars were sintered at 1100 °C for 12 h. Data for *x* = 0.05 are taken from ref. [18]. The three perovskites show a semiconductor-like behavior with an anomaly around 400 °C; this anomaly matches with the oxygen lost, observed in the thermal analysis curve. The maximum conductivity values at 850 °C for SrCo_1−*x*_Nb*_x_*O_3−*δ*_ (*x* = 0.05, 0.10 and 0.15) are σ = 46.5, 48.8, and 19.7 S∙cm^−1^, respectively. There is a clear decrease in the electrical conductivity for *x* = 0.15 with respect to *x* = 0.05 and 0.1 which could be related to the observed impurity of the hexagonal phase (Figure 1) and could contribute to hinder the electronic conductivity. The σ values for SrCo_0.9_Nb_0.1_O_3−*δ*_ are comparable to those of the most widely used derivative of SrCoO_3−*δ*_, such as Ba_0.5_Sr_0.5_Co_0.8_Fe_0.2_O_3−*δ*_ (BSCF) showing σ = 35 S·cm^−1^ at 850 °C [5,24] and SrCo_0.8_Fe_0.2_O_3−*δ*_ (SCF), chosen for its high oxygen fluxes and favorable performance for oxygen reduction.

### 3.5. Chemical Compatibility

**Figure 7:**
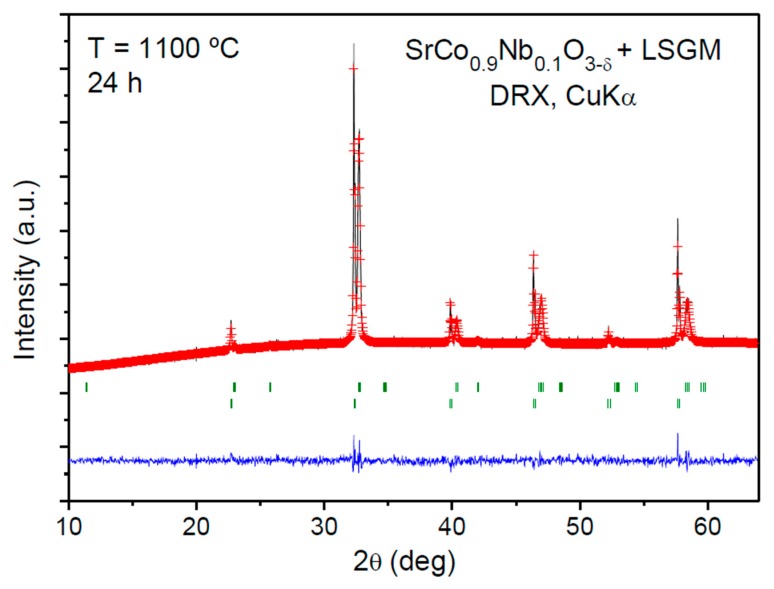
Rietveld-refined XRD profiles of a mixture of SrCo_0.9_Nb_0.1_O_3−*δ*_ and La_0.8_Sr_0.2_Ga_0.83_Mg_0.17_O_3−*δ*_ (LSGM) after thermal treatment at 1100 °C for 24 h in air.

The chemical compatibility between SrCo_1−*x*_Nb*_x_*O_3−_*_δ_* (*x* = 0.10, 0.15) and the electrolyte (LSGM) was checked by grinding both materials in equal amounts and heating them at 1100 °C for 24 h. Figure 7 illustrates the Rietveld-refined XRD profile of the SrCo_0.9_Nb_0.1_O_3−_*_δ_* calcined product, which consists of a mixture of both unchanged perovskite phases. This is essential for a good performance of these materials when they are tested in single-cells. The same result was obtained for SrCo_0.85_Nb_0.15_O_3−_*_δ_*.

### 3.6. Fuel-Cell Tests

**Figure 8:**
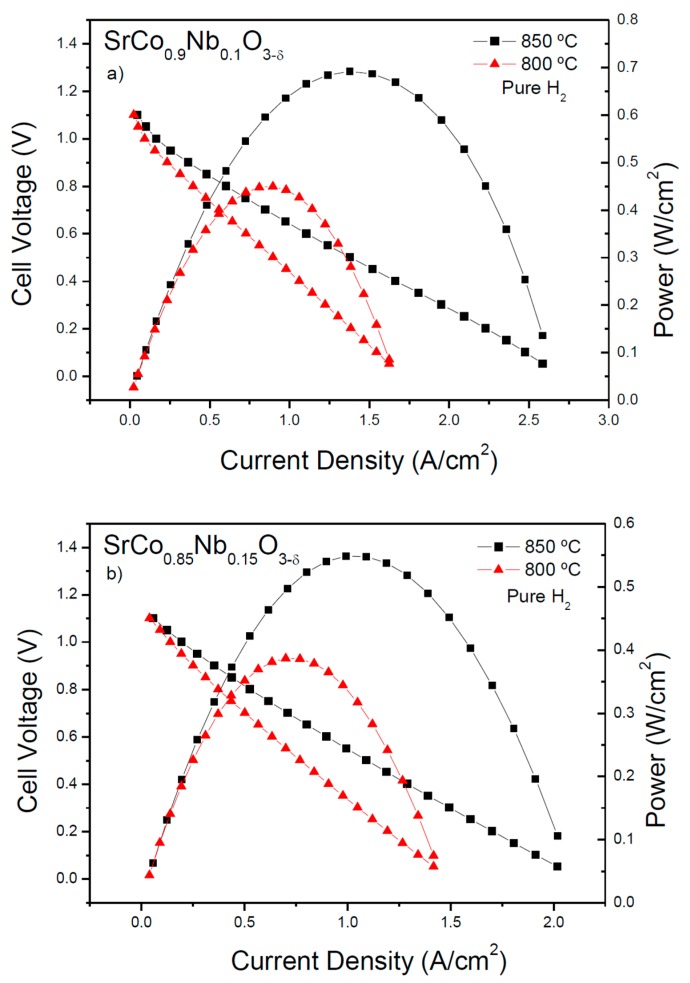
Cell voltage (left axis) and power density (right axis) as a function of the current density for the test cell with (**a**) the configuration SMFO/LDC/LSGM/SrCo_0.9_Nb_0.1_O_3−*δ*_ and (**b**) SMFO/LDC/LSGM/SrCo_0.85_Nb_0.15_O_3−*δ*_ in pure H_2_ measured at T = 800 and 850 °C.

The performance of SrCo_1−*x*_Nb*_x_*O_3−*δ*_ (*x* = 0.10 and 0.15) as cathode materials was tested in single cells in an electrolyte-supported configuration using a 300-µm-thick LSGM electrolyte. Figure 8a,b illustrate the cell voltage and power density as a function of the current density at 800 and 850 °C for the test cells fed with pure H_2_; the cathode materials worked in air atmosphere. The maximum power densities generated by the cells for the SrCo_0.90_Nb_0.1_O_3−*δ*_ and SrCo_0.85_Nb_0.15_O_3−*δ*_ cathodes are 693 and 550 mW∙cm^−2^ at 850 °C, respectively. If we compare these output powers with that reported for *x* = 0.05 in ref. [18] (595 mW∙cm^−2^ at 850 °C), we can appreciate a relationship between the electrical conductivity and the performance of these materials as cathodes.

The reasonably good performance is related to the electrical conductivities promoted by the 3C perovskite arrangement, consisting of corner-sharing octahedra forming a three-dimensional array where Co-O-Co paths favor the overlap between Co 3*d* and O 2*p* orbitals. As well, the presence of oxygen vacancies, well supported by this perovskite superstructure, favors oxygen diffusion; both properties are expected for a MIEC cathode. It is worth comparing these performances with measurements with the same electrolyte and related cathode materials. For example, we can mention a similar study with SrCo_0.95_Sb_0.05_O_3−*δ*_ as a cathode and the same electrolyte (LSGM) [17], where a maximum power density of 600 mW/cm^2^ at 850 °C was obtained.

## 4. Conclusions

The effect of Nb doping in the SrCo_1−_*_x_*Nb*_x_*O_3−_*_δ_* (*x* = 0.1 and 0.15) system has been evaluated. The introduction of Nb into the crystal structure promotes the formation of a tetragonal superstructure with doubled *c*-axis, in a 3C configuration where CoO_6_ octahedra share vertices across the three crystallographic directions. Therefore, the unwanted 2H hexagonal SrCoO_3−_*_δ_* polytype was avoided by doping with 10% and 15% Nb at the Co positions of the perovskite. NPD data unveil interesting features about the crystal structure of the *x* = 0.1 material: the refinement of oxygen occupancies shows a long-range ordering of the oxygen vacancies (concentrated at O3 sites) that accounts for the formation of the tetragonal superstructure. The large displacement factors of O3 suggest a significant ionic mobility, whereas the flattened ellipsoids observed for fully occupied O1 and O2 atoms imply strongly covalent Co-O-Co bonds, with degrees of freedom perpendicular to the bonding direction and meagre participation in the oxygen diffusion process. The electrical conductivity for both samples seems to be enough to deliver a good performance in test cells, yielding reasonable power densities with pure H_2_ as a fuel. This result relies on the association of determining factors such as the catalytic activity of Co ions and the favorable 3C crystal structure, yielding electronic and ionic conductivities arising from the presence of oxygen vacancies, corresponding to a MIEC electrode. Finally, an excellent chemical compatibility with the electrolyte LSGM is also observed.

## Figures and Tables

**Table 1:** Unit-cell parameters and discrepancy factors after the Rietveld refinements of the samples studied by X-ray diffraction (XRD) at room temperature. Values for *x* = 0.05 are taken from ref. [18], reprinted and adapted from [18], with permission from © 2014 Elsevier.

**SrCo_1−*x*_Nb*_x_*O_3−*δ*_**	***x* = 0.05**	***x* = 0.1**	***x* = 0.15**
a (Å)	3.8574(5)	3.8621(4)	3.8928(6)
c (Å)	7.743(1)	7.748(1)	7.7807(2)
V (Å)^3^	115.22(3)	115.56(2)	118.30(4)
**Reliability Factors**			
R_p_	2.74	2.81	4.55
R_wp_	3.56	3.78	5.87
R_exp_	2.76	2.71	4.57
χ^2^	1.67	1.94	1.65
R_Bragg_ (%)	8.82	7.78	14.6

**Table 2:** Unit-cell, positional, displacement parameters and selected atomic distances (Å) for SrCo_0.9_Nb_0.1_O_3−*δ*_ (compared with those for SrCo_0.95_Nb_0.05_O_3−_*_δ_* taken from ref. [18]), reprinted and adapted from [18], with permission from © 2014 Elsevier, defined in the tetragonal P*4*/*mmm* (n° 123) space group, Z = 2, from neutron power diffraction (NPD) data at 25 °C.

**SrCo_1−*x*_Nb*_x_*O_3−*δ*_**	***x* = 0.05**	***x* = 0.1**
a (Å)	3.85477(4)	3.85929(6)
c (Å)	7.7420(1)	7.7455(2)
V (Å)^3^	115.041(3)	115.362(4)
**Sr 2*h* (½, ½, *z*)**		
z	0.2585(3)	0.2564(4)
B_iso_ (Å^2^)	1.32(3)	1.15(3)
f_occ_	1.00	1.00
**(Co,Nb)1 1*a* (0, 0, 0)**		
B_iso_ (Å^2^)	0.5(1)	0.3(1)
f_occ_ (Co,Nb)	0.982/0.018	1/0.00
**(Co,Nb)2 1*b* (0, 0, ½)**		
B_iso_ (Å^2^)	1.1(1)	1.8(2)
f_occ_ (Co,Nb)	0.918/0.082	0.911/0.089
**O1 2*f* (½, 0, 0)**		
B_eq_ (Å^2^)	0.94(5)	1.37
β_11_ *	-	19(0)
β_22_	-	358(37)
β_33_	-	78(8)
f_occ_	0.96(1)	0.999(1)
**O2 2*g* (0, 0, *z*)**		
z	0.7643(6)	0.7624(5)
B_eq_ (Å^2^)	2.7(2)	1.88
β_11_	-	425(38)
β_22_	-	425(38)
β_33_	-	24(6)
f_occ_	0.991(3)	1.00
**O3 2*e* (½, 0, ½)**		
B_eq_ (Å^2^)	3.2(1)	3.07
β_11_	-	544(39)
β_22_	-	672(63)
β_33_	-	82(12)
f_occ_	0.776(1)	0.829(1)
**Reliability factors**		
R_p_	4.26	2.22
R_wp_	5.55	2.94
R_exp_	3.5	1.85
χ^2^	2.51	2.52
R_Bragg_ (%)	2.74	2.34
**Distances (Å)**		
Co1-O1 (×4)	1.9274(2)	1.92964(3)
Co1-O2 (×2)	1.830(6)	1.840(3)
(Co,Nb)2-O2 (×2)	2.041(6)	2.032(3)
(Co,Nb)2-O3 (×4)	1.9274(2)	1.92964(3)

* Anisotropic displacement factors (×10^4^); β_12_ = β_13_ = β_23_ = 0.

## References

[1] Kendall K. (2016). High—Temperature Solid Oxide Fuel Cells for the 21st Century.

[2] Nagai T., Ito W., Sakon T.R. (2007). Relationship between Cation Substitution and Stability of Perovskite Structure in SrCoO_3–_*_δ_*-based Mixed Conductors. Solid State Ion..

[3] Deng Z.Q., Liu W., Chen C.S., Lu H., Yang W.S. (2004). Germanium and Iron Co-Substituted SrCoO_2.5+_*_δ_* as Oxygen Permeable Membrane. Solid State Ion..

[4] Zeng P., Ran R., Chen Z., Zhou W., Gu H., Shao Z., Liu S. (2008). Efficient Stabilization of Cubic Perovskite SrCoO_3−_*_δ_* by B-site Low Concentration Scandium Doping Combined with Sol–Gel Synthesis. J. Alloys Compd..

[5] Shao Z.P., Haile S.M. (2004). A High-Performance Cathode for the Next Generation of Solid-Oxide Fuel Cells. Nature.

[6] Zhou W., Ran R., Shao Z. (2009). Progress in understanding and development of Ba_0.5_Sr_0.5_Co_0.8_Fe_0.2_O_3−*δ*_-based cathodes for intermediate-temperature solid-oxide fuel cells: A review. J. Power Sources.

[7] Tunney J.J., Post M.L., Du X., Yang D. (2002). Temperature Dependence and Gas-Sensing Response of Conduction for Mixed Conducting SrFe_y_Co_z_O_x_ Thin Films. J. Electrochem. Soc..

[8] Svarcova S., Wiik K., Tolchard J., Bouwmeester H.J.M., Grande T. (2008). Structural Instability of Cubic Perovskite Ba*_x_*Sr_1−_*_x_*Co_1−_*_y_*Fe*_y_*O_3–_*_δ_*. Solid State Ion..

[9] Bucher E., Egger A., Caraman G.B., Sitte W. (2008). Stability of the SOFC Cathode Material (Ba,Sr)(Co,Fe)O_3−*δ*_ in CO_2_-Containing Atmospheres. J. Electrochem. Soc..

[10] Deng Z.Q., Yang W.S., Liu W., Chen C.S. (2006). Relationship between transport properties and phase transformations in mixed-conducting oxides. J. Solid State Chem..

[11] Takeda Y., Kanno R., Takada T., Yamamoto O., Takano M., Bando Y. (1986). Phase relation and oxygen-non-stoichiometry of Perovskite-like Compound SrCoO_x_ (2.29 < *x* > 2.80). Z. Anorg. Allg. Chem..

[12] De la Calle C., Aguadero A., Alonso J.A., Fernández-Díaz M.T. (2008). Correlation between reconstructive phase transitions and transport properties from SrCoO_2.5_ brownmillerite: A neutron diffraction study. Solid State Sci..

[13] Li Y., Kim Y.N., Cheng J., Alonso J.A., Hu Z., Chin Y., Takami T., Fernández-Díaz M.T., Lin H.J., Chen C.T. (2011). Oxygen-Deficient Perovskite Sr_0.7_Y_0.3_CoO_2.65−_*_δ_* as a Cathode for Intermediate-Temperature Solid Oxide Fuel Cells. Chem. Mater..

[14] Liu T., Li Y., Goodenough J.B. (2012). Sr_0.7_Ho_0.3_CoO_3−*δ*_ as a Potential Cathode Material for Intermediate-Temperature Solid Oxide Fuel Cells. J. Power Sources.

[15] Aguadero A., Pérez-Coll D., Alonso J.A., Skinner S.J., Kilner J. (2012). A new family of Mo-doped SrCoO_3−*δ*_ perovskites for application in reversible solid state electrochemical cells. Chem. Mater..

[16] Wang S.F., Hsu Y.F., Yeh C.T., Huang C.C., Lu H.C. (2012). Characteristics of SrCo_1−_*_x_*Sn*_x_*O_3−_*_δ_* cathode materials for use in solid oxide fuel cells. Solid State Ion..

[17] Aguadero A., Alonso J.A., Perez-Coll D., de la Calle C., Fernández-Díaz M.T., Goodenough J.B. (2010). SrCo_0.95_Sb_0.05_O_3−*δ*_ as cathode material for high power density solid oxide fuel cells. Chem. Mater..

[18] Cascos V., Martínez-Coronado R., Alonso J.A. (2014). New Nb-doped SrCo_1−*x*_Nb*_x_*O_3−*δ*_ perovskites performing as cathodes in solid-oxide fuel cells. Int. J. Hydrog. Energy.

[19] Rietveld H.M.A. (1969). Profile refinement method for nuclear and magnetic structures. J. Appl. Crystallogr..

[20] Rodríguez-Carvajal J. (1993). Recent advances in magnetic structure determination by neutron powder diffraction. Physica B.

[21] Martínez-Coronado R., Alonso J.A., Aguadero A., Fernández-Díaz M.T. (2012). Optimized energy conversion efficiency in solid oxide fuel cells implementing SrMo_1−*x*_Fe*_x_*O_3−*δ*_ perovskites as anodes. J. Power Sources.

[22] Shannon R.D. (1976). Revised effective ionic radio and systematic studies of interatomic distances in halides and chalcogenides. Acta. Crystallogr. A.

[23] Cascos V., Troncoso L., Alonso J.A. (2015). New families of M^n+^-doped SrCo_1−*x*_M*_x_*O_3−*δ*_ perovskites performing as cathodes in solid-oxide fuel cells. Int. J. Hydrog. Energy.

[24] Chen Z., Ran R., Zhou W., Shao Z., Liu S. (2007). Assessment of Ba_0.5_Sr_0.5_Co_1−*y*_Fe*_y_*O_3−*δ*_ (y = 0.0–1.0) for prospective application as cathode for IT-SOFCs or oxygen permeating membrane. Electrochim. Acta.

